# The intrafollicular concentration of leptin as a potential biomarker to predict oocyte maturity in in-vitro fertilization

**DOI:** 10.1038/s41598-022-23737-1

**Published:** 2022-11-15

**Authors:** Kun-Jing Hong, Jun-Jie Lin, Liang-Hua Lin, Tsung-Hsuan Lai

**Affiliations:** 1 Cathay Medical Research Institute, Cathay General Hospital, New Taipei City, 22174 Taiwan; 2 Department of Obstetrics and Gynecology, Assisted Reproductive Center, Cathay General Hospital, No. 280, Sec. 4, Renai Rd., Daan Dist., Taipei, 10693 Taiwan; 3 Department of Oral Hygiene Care, Ching Kuo Institute of Management and Health, Keelong, Taiwan; 4 School of Medicine, Fu-Jen Catholic University, No. 510, Zhongzheng Rd., Xinzhuang Dist., New Taipei City, 242062 Taiwan

**Keywords:** Developmental biology, Biomarkers, Endocrinology

## Abstract

Oocyte maturity is critical to the development potential of the embryo and pregnancy outcomes in natural and in-vitro fertilization (IVF). In IVF, oocyte maturity is typically evaluated using morphological criteria, although such assessment remains highly subjective. To identify reliable biomarkers of oocyte maturity, this study investigates the relationship between follicular cytokine concentrations and oocyte maturity in IVF patients with different ovarian reserves. In this prospective study, follicular fluid was collected during oocyte retrieval and the concentrations of cytokines involved in ovarian folliculogenesis were determined. Follicular fluid cytokine concentrations were compared between participants in three groups according to serum anti-Mullerian hormone (AMH) concentration, as follows: low AMH, < 2 ng/mL; normal AMH, 2–5 ng/mL; and high AMH, > 5 ng/mL. Pearson's correlation coefficient analysis showed that the number of mature oocytes correlated positively and strongly with serum AMH level (r = 0.719; p < 0.01). The leptin concentration in follicular fluid was significantly higher in women with normal AMH level than in those with low or high levels. ROC curve analysis showed that the follicular fluid levels of leptin (area under ROC curve, 0.829; 95% confidence interval, 0.659–0.998; p < 0.01) and SCF (area under ROC curve, 0.706; 95% confidence interval, 0.491–0.921; p = 0.087) were the best predictors of oocyte maturity. At an optimal cut-off value of 16 ng/mL, leptin had positive predictive value (sensitivity) up to 70% and negative predictive value (specificity) of 91% for indicating oocyte maturity. The concentration of leptin in follicular fluid is closely related to ovarian reserve and may serve as a biomarker to predict oocyte maturity.

## Introduction

Folliculogenesis is a complex series of biological events that includes the coordination of primordial follicle recruitment, granulosa/theca cell proliferation, oocyte maturation, and steroidogenesis^1^. Each of these stages involves a paracrine dialogue between the oocyte and granulosa/theca cell layers, principally mediated by the interactions of hormones and cytokines^2–4^. Cytokines are small, soluble signaling proteins best known for their immunoregulatory properties, but increasingly recognized as growth factors governing cell proliferation, differentiation, function, and fate^5,6^. They include interleukins (ILs), colony stimulating factors (CSFs), tumor necrosis factors (TNFs), transforming growth factors (TGFs), and other peptide hormones produced by a variety of cell types. Additionally, cytokines are keys to reproductive success, creating an immune-permissive, embryotrophic environment that supports gametogenesis, fertilization, implantation, embryo development, and fetal growth^6–10^.

The importance of follicular cytokines in folliculogenesis is increasingly recognized, although our understanding of their precise roles and interactions remains limited. Specific signaling pathways/paracrine dialogues have been identified for a number of cytokines^11^, although many of the supporting data are fragmented and relate to specific aspects of ovarian function, follicular development, and oocyte maturity. For example, interleukins and cytokines of the TGF-β family participate in intercellular communication between the oocyte and its surrounding granulosa cells, as well as in the regulation of follicle survival and apoptosis^7,12–14^. Ovarian immune cells such as macrophages and lymphocytes also secrete cytokines, including interferon γ (IFN-γ), TNF-α, granulocyte colony stimulating factor (G-CSF), IL-1, IL-6 and IL-8, monocyte chemoattractant protein 1 (MCP-1), and granulocyte macrophage CSF, all of which have been implicated in oocyte development^15,16^.

The capacity of ovaries to produce viable eggs, known as ovarian reserve, depends upon the response to ovarian stimulation, follicular development, oocyte quantity, and even oocyte quality in in-vitro fertilization (IVF) cycles^17^. Ovarian reserve tests designed to determine oocyte quantity and quality include assessments of serum basal Follicle-stimulating hormone (FSH), estradiol, the antral follicle count (AFC), and anti-Mullerian hormone (AMH). These measures are used to predict ovarian response and pregnancy rate. AMH is suggested to be a better predictor of ovarian reserve and the possibility of pregnancy than is age, basal FSH, estradiol, and AFC, both by nature and in assisted reproduction^18,19^. A serum AMH level under 0.7 ng/mL indicates poor ovarian reserve and is associated with significantly reduced natural pregnancy in the general population^20^.

Successful IVF requires the production of mature oocytes in response to ovarian stimulation with adequate gonadotropins. Because a mature oocyte has greater potential for fertilization and embryo development, greater production of mature oocytes can result in higher rates of pregnancy and live births^21^. In clinical practice, oocyte maturity is typically evaluated using morphological criteria, despite evidence that this assessment method may not be optimal^22^. Although the reliability of morphological assessment can be improved by denuding the oocyte, this technique remains highly subjective, is open to inaccuracies, and renders the oocyte incapable of further development^23,24^. Previous studies have shown that the microenvironment of the follicular fluid (FF) is highly related to oocyte maturity^25^. Thus, the FF cytokine profile may be an indicator of oocyte maturity, although this relationship has not yet been established. Because cytokines mediate many stages of folliculogenesis, it is reasonable to expect that their expression levels may be indicators of oocyte viability, fertilization potential, and embryo development. In this case, follicular cytokine levels could be of diagnostic or prognostic value in assisted reproduction; analysis of the cytokine profile of FF collected at the time of oocyte retrieval would offer a non-invasive analytical strategy for assessing the developmental potential of an oocyte.

Whether the level of ovarian reserve is associated with a particular cytokine profile in the preovulatory leading follicle is unknown. We conduct this study to explore the possible roles of follicular cytokines as predictors for oocyte maturation in the IVF patients with low, normal, and high ovarian reserve.

## Methods

### Study design and patient recruitment

**Figure 1:**
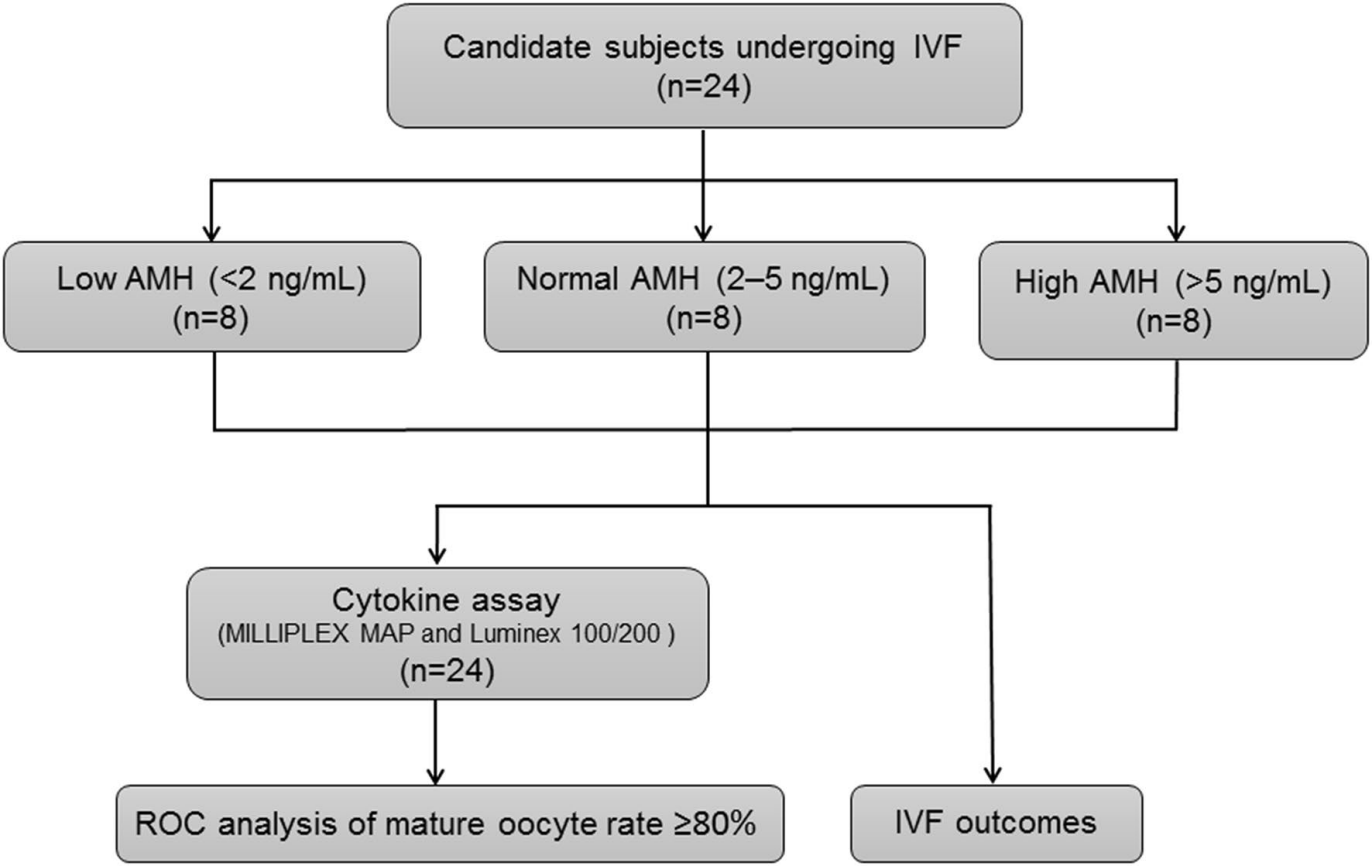
Flowchart of study design. 24 infertile women under IVF treatment were recruited and divided into three groups (low ovarian reserve, < 2 ng/mL; normal ovarian reserve, 2–5 ng/mL; and high ovarian reserve, > 5 ng/mL) according to ovarian reserve (serum AMH level). Cytokine analyses were performed on these three groups.

This is a prospective study. The study was designed to evaluate the FF cytokine contribution to mature oocytes and was presented in Fig. 1. All research was performed in accordance with the relevant guidelines and regulations. All the 24 selected patients were recruited and no one quit in this study. Human follicular fluid samples were collected from women undergoing IVF treatment. Inclusion criteria were as follows: (1) age < 45 years, (2) serum FSH < 15 mIU/mL on cycle days 2 or 3, (3) first egg retrieval cycle, (4) ovulation stimulation in response to the GnRH antagonist protocol, (5) no chromosomal abnormalities, and (6) 18.0 ≤ BMI ≤ 35.0 kg/m^2^. Exclusion criteria were as follows: (1) ovarian pathologies (endometrioma, cysts, teratomas, benign ovarian tumors); (2) ovarian cancers; (3) repeated egg retrieval; (4) ovulation stimulation with a non-GnRH antagonist protocol; (5) ovarian failure (AMH < 0.01 ng/ml); (6) BMI < 18.0 kg/m^2^ or BMI > 35.0 kg/m^2^; (7) sexually transmitted diseases; (8) poor ovarian responder (< 3 eggs retrieved); and (9) male infertility.

The serum AMH of each patient was measured before the IVF procedure. The patients were assigned to three groups according to their serum AMH level: low ovarian reserve, < 2 ng/mL; normal ovarian reserve, 2–5 ng/mL; and high ovarian reserve, > 5 ng/mL.

### Ovarian stimulation protocol

Ovarian stimulation was induced in all participants using the GnRH antagonist protocol. A controlled ovarian stimulation (COS) protocol with human recombinant FSH (Gonal-F, Merck Serono) and highly purified hMG-HP (Menopur, Ferring) was initiated on menstrual cycle days 2 or 3 at a variable dose depending on age, body weight, AMH and AFC, ranging from rFSH 150 to 300 IU and hMG 75 to 225 IU daily. The dose was adjusted during ovarian stimulation based on the ovarian response to the starting dose. Pituitary suppression was achieved by daily subcutaneous (SC) administration of 0.25 mg of cetrorelix acetate (Cetrotide, Merck Serono) when at least one follicle exceeded a mean diameter of 14 mm or when estrogen levels (E2) were over 500 pg/mL. Ovulation was triggered by dual triggers with SC administration of 3000 U of hCG (Ovidrel, Merck-Serono) and 0.2 mg of GnRH agonist (Decapeptyl, MSD) when at least 3 leading follicles were > 18 mm. Transvaginal oocyte retrieval was performed at 35–37 h after dual trigger. The information of ovarian stimulation is summary in Table 1.

**Table 1:** The baseline parameters and IVF outcomes of the patients.

Variables	Mean ± SD
No. of cases	24
Age (years)	37.2 ± 4.8
Serum AMH (ng/mL)	4.17 ± 3.03
AFC	12.5 ± 5.6
AMH in FF (ng/mL)	2.27 ± 1.07
BMI (kg/m^2^)	20.97 ± 2.66
Basal E2 (pg/mL)	28.26 ± 16.88
Basal LH (mIU/mL)	5.24 ± 2.98
Basal P4 (ng/mL)	0.22 ± 0.17
Basal FSH (mIU/mL)	5.78 ± 2.72
Total rFSH dose (IU)	1759.09 ± 422.83
Total hMG dose (IU)	668.18 ± 592.32
Duration of ovarian stimulation (day)	9.5 ± 0.9
E2 on trigger day (pg/mL)	3191.10 ± 2454.16
LH on trigger day (mIU/mL)	2.70 ± 2.23
P4 on trigger day (ng/mL)	0.78 ± 0.48
No. of total oocyte retrieved	12.0 ± 8.6
No. of MII oocytes	9.2 ± 5.9
Oocyte maturation rate (%)	78.42 ± 23.91
No. of embryo transfer	2.7 ± 0.9
Fertilization rate (%)	73.22 ± 21.66
Cleavage embryos (%)	75.00 ± 18.85
Blastocyst embryos (%)	25.00 ± 18.85
Implantation rate (%)	37.25 ± 38.77
Ongoing pregnancy rate (%)	55.26 ± 15.51

*AMH* anti-Mullerian hormone, *FF* follicular fluid, *BMI* body mass index, *MII* metaphase II, *AFC* antral follicle count, *E2* estradiol, *LH* luteinizing hormone, *P4* progesterone, *FSH* follicle stimulating hormone, *rFSH* recombinant follicle stimulating hormone, *hMG* human menopausal gonadotropin.

### Sample collection

FF samples were collected and pooling from three preovulatory follicles in each patient during oocyte retrieval. Aspiration of the oocytes and FF was performed under transvaginal ultrasound guidance. The FF collection procedure was proceeded very carefully to avoid blood contamination, and the FF was obtained without flushing by culture medium, minimizing the wash medium left in the tube that would dilute the FF during collecting. If blood contamination occurred, that FF sample was discarded. Otherwise, the obtained FF was then further centrifuged at 1000×*g* for 3 min to remove possibly contaminated blood cells or cell debris. The collected FF sample was aliquoted to the tubes and stored at − 80 °C for further analysis.

### Serum hormone analysis

Serum samples were collected on menstrual cycle day 2 and the trigger day. The serum AMH level of each patient was measured before the IVF cycle. The concentrations of hormones including AMH (Elecsys AMH), FSH (Elecsys FSH), LH (Elecsys LH), and E2 (Elecsys Estradiol III) were measured using a chemiluminescence-based immunometric assay on cobas e 601 (UZB) analyzers. The concentration of progesterone (Elecsys Progesterone III) was detected by cobas e 411 (IVI) analyzers. All hormone assays were performed as per the manufacturer’s instructions and the limit of quantitation was described in Supplementary Table 1.

### Measurement of follicular cytokines

Using information in the literature, we selected the following 29 candidate cytokines related to folliculogenesis and oocyte maturity for assessment using MILLIPLEX MAP (Merck EMD Millipore, Billerica, MA, United States) and Luminex 100/200 (Luminex Corporation): PDGF-AA, PDGF-AB/BB, IFNr, IL-15, IL-1B, IL-6, IL-7, IL-8, MCP-1, TNFa, EGF, angiopoitin-2, BMP-9, endothelin-1, FGF-1, HB-EGF, VEGF-C, VEGF-D, FGF-2, VEGF-A, leptin, sFasL, sFas, prolactin, SCF, OPN, TGFβ1, TGFβ2, and TGFβ3. A 6-point standard curve was generated and was used to determine the concentration of a solution. All serum samples quantified in reference to respective standards. The concentrations were determined by comparison to standards and are reported as averages. The detection range for each cytokine showed the in Supplementary Table 1.

### Statistical analyses

Clinical data were collected from medical charts and are presented as the mean ± standard deviation (SD) for quantitative variables. Data were analyzed using non-parametric Mann–Whitney U test for comparison of mean values, with p < 0.05 considered significant. FF cytokine/chemokine concentrations were analyzed for association with oocyte maturation and serum AMH concentration using SPSS 16.0 software (SPSS Inc., Chicago). Correlations between oocyte maturity and cytokine/chemokine concentrations were analyzed using Pearson correlation coefficients. Receiver operating characteristic (ROC) curves were used to assess the value of FF cytokine/chemokine concentration for predicting high quality oocytes. An oocyte maturation rate ≥ 80%^26^ was defined as AUC = 1. The area under a ROC curve was used to determine the probability of accurately distinguishing high-quality oocytes from the others.

### Ethics approval and consent to participate

The study was approved by the Ethics Committee of Cathay General Hospital, Taipei, Taiwan, and written informed consent was obtained from all patients (IRB No.: CGH-P106065).

## Results

### Demographic characteristics

**Figure 2:**
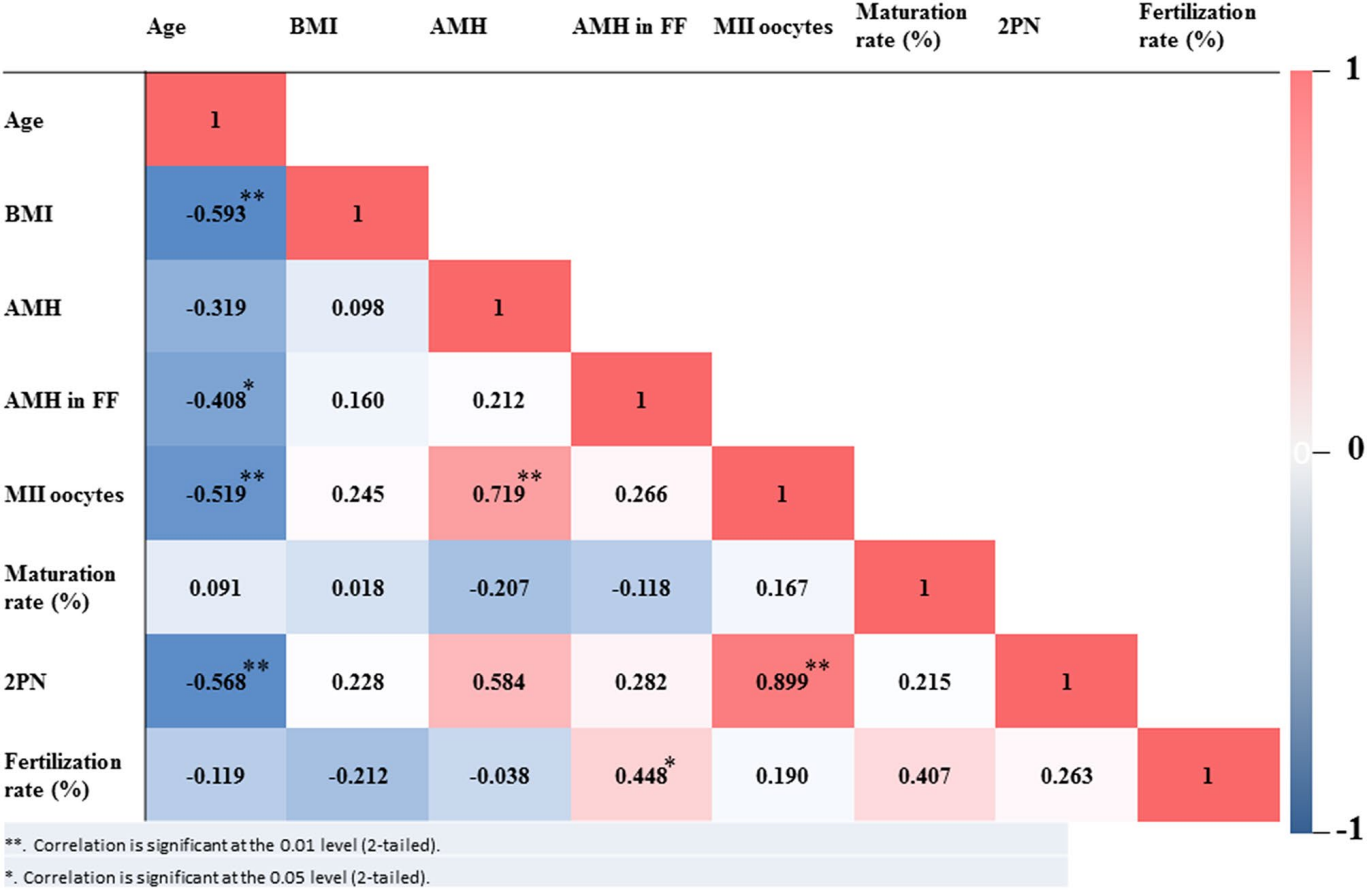
Heatmap showing the correlation coefficients between characteristics and ovarian response of participants. The heatmap was created in Excel (Microsoft 365) by using conditional formatting. Dark red, high correlation (near 1); dark blue, high anti-correlation (near − 1); *p < 0.05; **p < 0.01.

The clinical characteristics and IVF outcomes of the 24 participants are summarized in Table 1. The Pearson correlation coefficient heatmap of the parameters (Fig. 2) indicated that the highest correlation was between MII oocytes and 2PN (r = 0.899; p < 0.01). Serum AMH concentration correlated highly with MII oocytes (r = 0.719; p < 0.01). The fertilization rate also correlated positively with FF-AMH and oocyte maturation (r = 0.448; p < 0.05). A negative correlations was observed between age and FF-AMH level (r =  − 0.408; p < 0.05), MII oocytes (r =  − 0.519; p < 0.01) and 2PN (r =  − 0.519; p < 0.01).

Clinical characteristics and IVF outcomes were compared between groups according to serum AMH concentration (Table 2). We observed no difference in BMI, basal E2, basal LH, basal P4, basal FSH, FF-AMH, total gonadotropin doses, duration of ovarian stimulation, E2/LH/P4 on trigger day, fertilization rate or pregnancy rate between the three groups. The total number of oocytes and the number of MII oocytes significantly increased with increasing ovarian reserve. The oocyte maturation rate differed significantly between the groups (serum AMH: low, 76.3 ± 37.01%; normal, 87.7 ± 10.41%; high, 71.3 ± 15.40%, p < 0.05). We also found that the serum FSH concentration was significantly higher in the low AMH group (Table 2).

**Table 2:** The characteristics and IVF outcomes of the study population grouping by AMH concentration.

Ovarian reserve	Low (AMH < 2.0) (n = 8)	Normal (2.0 < AMH < 5.0) (n = 8)	High (AMH > 5.0) (n = 8)
Age (years)	40.1 ± 2.4*	36.1 ± 4.7	35.4 ± 5.6
Serum AMH (ng/mL)	1.42 ± 0.43**	3.13 ± 0.44	7.97 ± 1.85**
AMH in FF (ng/mL)	2.11 ± 1.01	2.10 ± 1.31	2.59 ± 0.91
BMI (kg/m^2^)	20.11 ± 1.72	21.52 ± 2.58	21.27 ± 3.51
AFC	8.0 ± 3.2	12.5 ± 4.3	18.3 ± 4.4*
Basal E2 (pg/mL)	36.35 ± 19.56	23.73 ± 17.50	24.13 ± 11.65
Basal LH (mIU/mL)	4.84 ± 2.88	4.28 ± 2.40	6.49 ± 3.43
Basal P4 (ng/mL)	0.22 ± 0.16	0.19 ± 0.11	0.25 ± 0.23
Basal FSH (mIU/mL)	7.76 ± 3.16*	4.91 ± 1.00	4.55 ± 2.35
Total rFSH dose (IU)	1518.75 ± 390.23	1884.38 ± 428.86	1912.5 ± 366.66
Total hMG dose (IU)	984.38 ± 662.91	637.5 ± 556.94	287.5 ± 297.80
Duration of ovarian stimulation (day)	9.0 ± 0.5	9.9 ± 1.2	9.7 ± 0.5
E2 on trigger day (pg/mL)	1693.19 ± 575.45	3511.5 ± 1655.78	4368.63 ± 3528.52
LH on trigger day (mIU/mL)	2.78 ± 1.96	2.84 ± 2.90	2.47 ± 1.98
P4 on trigger day (ng/mL)	0.49 ± 0.26	0.80 ± 0.32	1.06 ± 0.64
No. of total oocytes	4.3 ± 1.8**	10.8 ± 4.6	21.1 ± 7.2**
No. of MII oocytes	3.4 ± 2.4**	9.6 ± 4.7	14.6 ± 4.0
No. of 2 PN	2.9 ± 2.0*	7.5 ± 4.2	11.1 ± 3.9
Oocyte maturation rate (%)	76.25 ± 37.01	87.74 ± 10.4	71.28 ± 15.40*
Fertilization rate (%)	72.80 ± 34.42	74.80 ± 14.23	71.89 ± 9.82
Cleavage embryos (%)	100.00 ± 0.00	62.50 ± 0.00	60.00 ± 0.00
Blastocyst embryos (%)	0	37.50 ± 0.00	40.00 ± 0.00
D3 transfer (%)	100.00 ± 0.00	62.50 ± 0.00	60.00 ± 0.00
D5 transfer (%)	0	37.50 ± 0.00	40.00 ± 0.00
Fresh embryo transfer (%)	28.57 ± 0.00**	0	0
Frozen embryo transfer (%)	71.43 ± 0.00	100.00 ± 0.00	100.00 ± 0.00
Cycle cancelation (%)	0	0	0
Transfer No	3.43 ± 0.79*	2.12 ± 0.64	2.60 ± 0.89
Implantation rate (%)	23.61 ± 29.07	45.24 ± 45.86	43.75 ± 42.70
Cumulative live birth rate (%)	42.86 ± 0.00	50.00 ± 0.00	80.00 ± 0.00**
Leptin (ng/mL)	13.64 ± 6.52*	19.55 ± 4.88	12.61 ± 6.61*
SCF (ng/mL)	14.91 ± 8.64	18.57 ± 3.84	11.35 ± 4.97**
PDGF-AA (pg/mL)	335.12 ± 73.67**	156.06 ± 98.50	244.32 ± 165.64

Values are presented as mean ± SD.

*AFC* antral follicle count, *AMH* anti-mullerian hormone, *FF* follicular fluid, *BMI* body mass index, *E2* estradiol, *LH* luteinizing hormone, *P4* progesterone, *FSH* follicle-stimulating hormone, *MII* metaphase II, *2PN* two pronuclei, *rFSH* recombinant follicle stimulating hormone, *hMG* human menopausal gonadotropin, *SCF* stem cell factor, *PDGF-AA* platelet-derived growth factor AA.

Mann–Whitney U test, *mean p < 0.05; **mean p < 0.01: compared to normal group.

### Cytokine and chemokine concentrations

**Figure 3:**
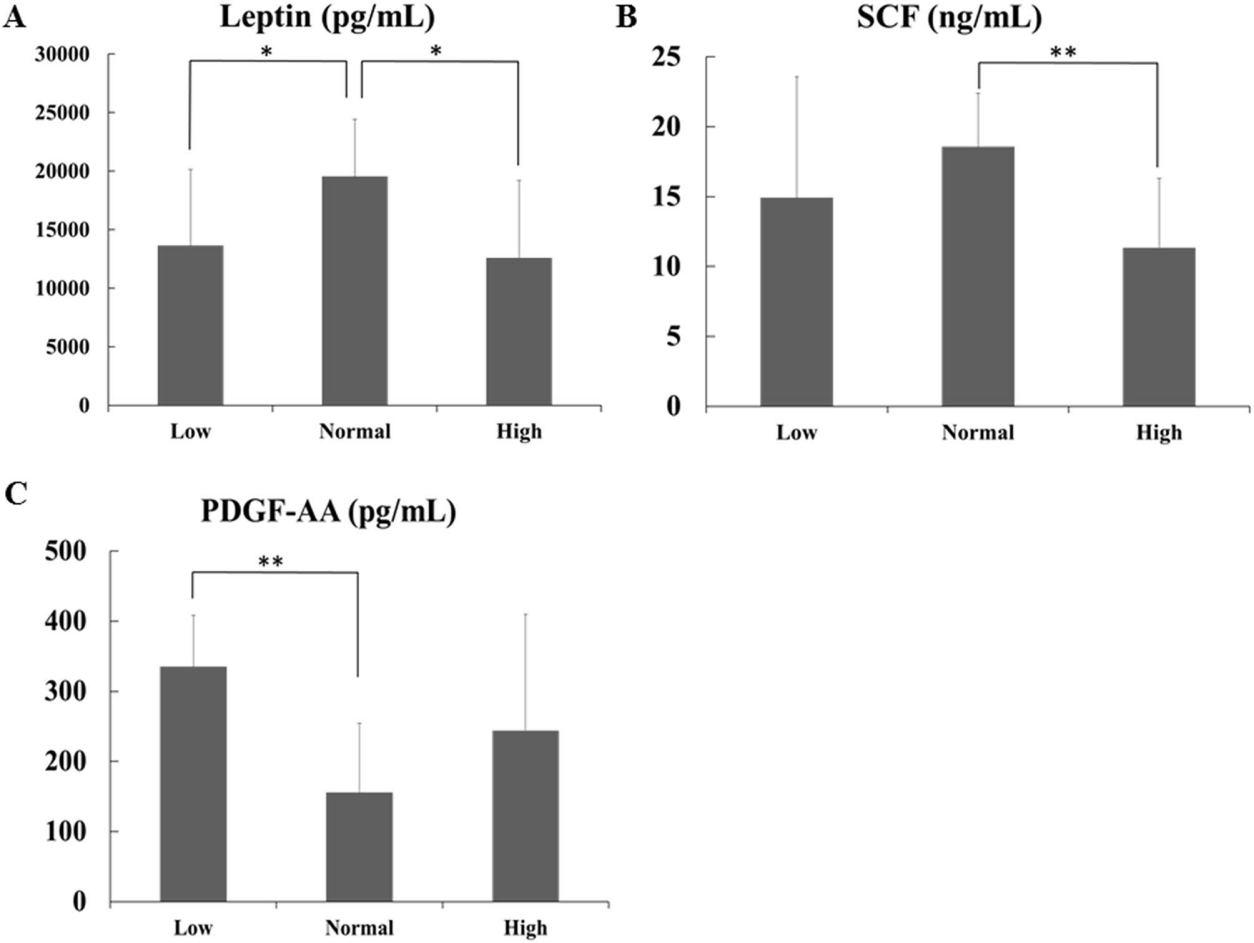
Significant differences in cytokine levels of follicular fluid according to AMH level. Cytokine concentrations (pg/mL) in follicular fluid collected during oocyte retrieval, including (**A**) leptin, (**B**) stem cell factor (SCF), and (**C**) platelet derived growth factor AA (PDGF-AA). Data are presented as the mean ± SD. Statistical analyses were performed using the Kruskal–Wallis test. *p < 0.05; **p < 0.01. Groups: low, low ovarian reserve (AMH < 2 ng/mL); normal, normal ovarian reserve (AMH 2–5 ng/mL); high, high ovarian reserve (AMH > 5 ng/mL).

Of the 29 cytokines/chemokines assessed in FF, 3 differed significantly between groups according to serum AMH level (Fig. 3). The leptin and concentration was significantly higher in the normal AMH group than in the low and high AMH groups (Fig. 3A). The normal AMH group also had higher SCF than did the high AMH group (Fig. 3B). PDGF-AA was significantly lower in the normal than in the low AMH group (Fig. 3C).

### Cytokines/chemokines in FF predictive of oocyte maturity

**Figure 4:**
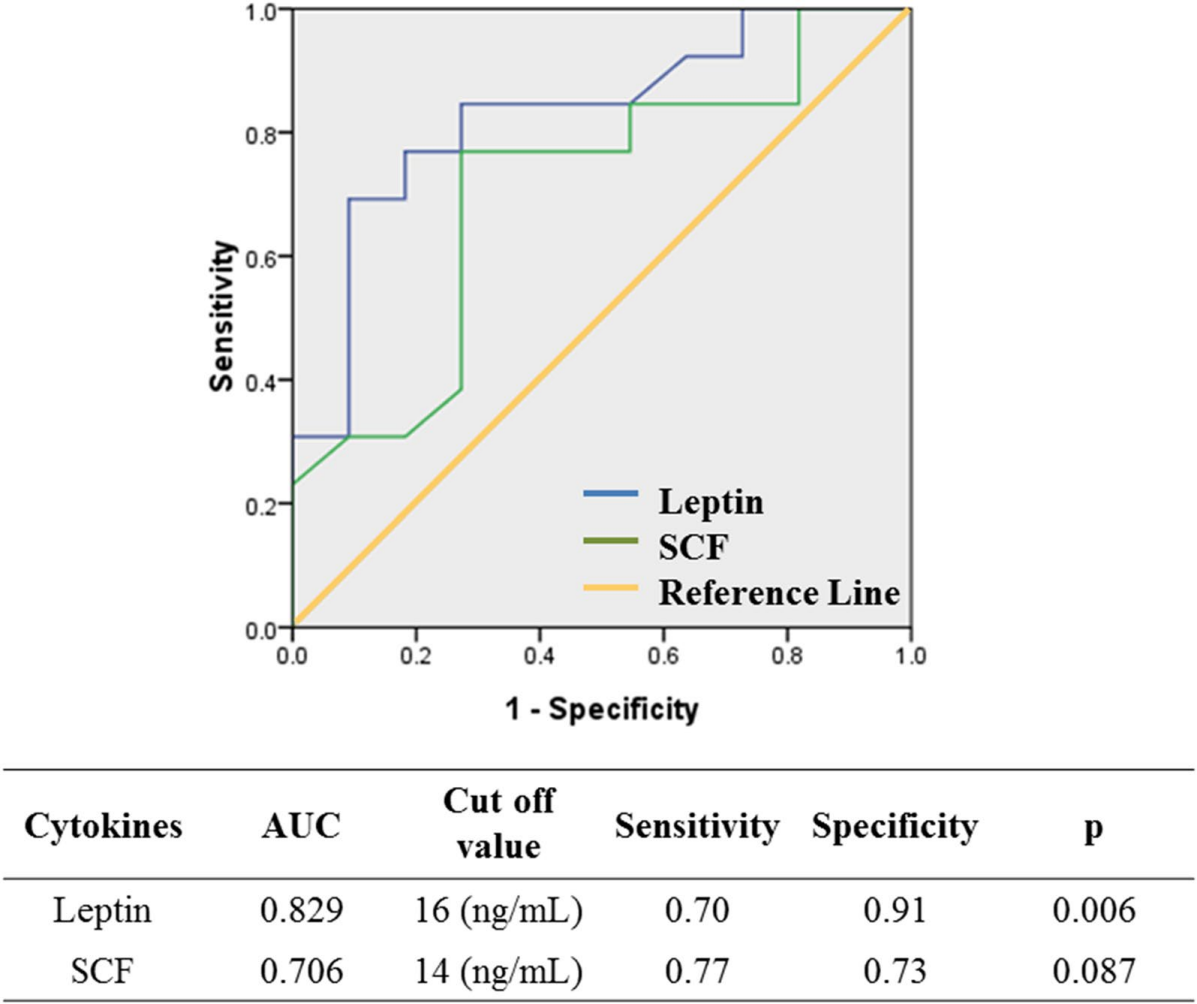
Receiver operating characteristics (ROC) analysis for predicting oocyte maturity according to follicular-fluid cytokine concentrations. The area under the ROC (SPSS output) was 0.829 for leptin [optimal cut-off value, 16 ng/mL; 95% confidence interval (CI), 0.658–0.998; p < 0.01) and 0.706 for SCF (optimal cut-off value, 14 ng/mL; 95% CI, 0.491–0.921; p = 0.087]. Leptin had positive predictive value (sensitivity) up to 70% and negative predictive value (specificity) of 91% for indicating oocyte maturity at its optimal cut-off value. An oocyte maturation rate ≥ 80% was defined as AUC = 1.

ROC analysis was used to determine whether any cytokine/chemokine levels in FF are predictive of oocyte maturity (Fig. 4). Because the proportion of oocytes that reach metaphase II (MII) was around 80% in human oocytes (27), an oocyte maturation rate ≥ 80% was defined as AUC = 1. From 29 cytokines/chemokines, we found that Leptin and SCF were the best predictors of oocyte maturity. The area under the ROC was 0.829 for leptin (optimal cut-off value, 16 ng/mL; 95% confidence interval [CI], 0.658–0.998; p < 0.01) and 0.706 for SCF (optimal cut-off value, 14 ng/mL; 95% CI, 0.491–0.921; p = 0.087). However, Leptin had the best positive predictive value (sensitivity) up to 70% and negative predictive value (specificity) of 91% for indicating oocyte maturity at its optimal cut-off value.

## Discussion

In this presenting study, we found that the number of mature oocytes correlated positively and strongly with AMH level (r = 0.719; p < 0.01). Moreover, the leptin concentration in follicular fluid was significantly higher in women with normal AMH level than in those with low or high levels. ROC curve analysis showed that the follicular fluid levels of leptin (area under ROC curve, 0.829; 95% confidence interval, 0.659–0.998; p < 0.01) and SCF (area under ROC curve, 0.706; 95% confidence interval, 0.491–0.921; p = 0.087) were the best predictors of oocyte maturity. At an optimal cut-off value of 16 ng/mL, leptin had the best power of positive predictive value (sensitivity) up to 70% and negative predictive value (specificity) of 91% for indicating oocyte maturity. Our findings concluded that the concentration of leptin in follicular fluid is closely related to ovarian reserve and oocyte maturity. Although a low number of patients includes in the study, this is the first research to indicate intrafollicular leptin concentration was significantly higher in women with normal AMH level than in those with low or high levels. We think our study results imply that intrafollicular leptin has a potential role as a biomarker to predict oocyte maturity. Further studies with larger samples should be conducted to make a solid conclusion.

Good-quality embryos are derived from good-quality mature oocytes. The follicular fluid is the medium in which make a communication loop between granulosa cells and oocytes. All regulatory molecules pass on in this microenvironment and ensure oocyte growth. As such, follicular components play a critical role in oocyte development and may be biomarkers of oocyte quality. We found that of the 29 FF cytokines/chemokines investigated in this study, leptin level was the strongest predictor of oocyte maturity. Leptin is a 167-amino-acid hormone secreted by fat cells in adipose tissue which influences reproduction via regulation of the hypothalamus–pituitary axis and ovarian function^27,28^. First discovered in cloned obese mice, leptin deficiency due to homozygous mutations was shown to result in extreme obesity and infertility^29^. Recent studies show that leptin is important in the regulation of fertility. Both high and low levels of leptin affect oocyte maturation in an animal model^30–32^, and serum leptin levels of normal-weight human females range from 16.7 to 20.4 ng/mL^33^. Leptin has been shown to promote oocyte maturation and embryo development in in-vitro culture of buffalo^31^ and sheep^32^.

We observed here that intrafollicular leptin levels were significantly lower in women with low and high ovarian reserve than in those with normal ovarian reserve. Lower leptin level correlated with low ovarian reserve and was the strongest predictor of oocyte maturation of the cytokines/chemokines assessed in this study. The 91% specificity and a 70% sensitivity exhibited by leptin are consistent with values reported by Llaneza-Suarez^34^. Menezes et al. report that oocyte quality is related to mitochondrial function and that the use of 10 ng/mL leptin maintains the mitochondrial integrity of oocytes^35^. These results indicate that oocyte maturation requires stimulation by leptin above a threshold concentration. We found that the presence of leptin in FF at a cutoff value of 16 ng/mL predicts oocyte maturity. Thus, intrafollicular leptin might serve as a biomarker to indicate oocyte maturity.

Given the important function of FF leptin, its regulation is of interest. Leptin mRNA is present in granulosa and theca cells, but not in oocytes, suggesting that leptin is produced elsewhere and then taken up by endocytosis. Some studies report that low levels of leptin are associated with ovarian failure, granulosa cell apoptosis, and gonadal function loss^36,37^. In the present study, we also observed leptin mRNA in granulosa cells. Although not significant, Leptin expression was highest among patients with normal ovarian reserve, although without significance (data not shown), with results similar to those shown in Fig. 3A.

Leptin can be detected in serum and follicle fluids. The level of leptin in serum correlated with the Body Mass Index (BMI) and the percentage of body fat. In our data, BMI of all subjects were not overweight (BMI > 30). Therefore, we focused on relationship between leptin concentration in follicular fluid and oocyte maturity. In the human ovary, leptin was produced by ovarian somatic cells^38^. In vitro studies indicated that leptin significantly increased the proportion of Porcine oocytes reaching MII^39^. The action of leptin on oocyte nuclear maturation was regulated by MAPK pathway and similar results were confirmed in mouse study^40^. These findings suggested MAPK pathway was essential communication between leptin and maturation of oocytes.

The level of AMH correlates with total number of oocytes retrieved and IVF success rate in previous studies^18–20^. As known, oocyte maturity is one of the main factors for IVF successful rate, not the total number of retrieved oocytes only. This is a preliminary report to address serum AMH level correlated to oocyte maturity. In the study, it is interesting that the number of mature oocytes correlated positively and strongly with serum AMH level (r = 0.719; P < 0.01), but not intrafollicular AMH level. The mechanism is unclear and we are going to conduct another study to explore this issue.

This study has several limitations. First, the small sample size may have affected the reliability of our results. Second, FF was collected from multiple individual follicles, so determining the pregnancy outcome of single embryo transfer derived from a mature oocyte is difficult. Further research is needed in larger cohorts to investigate FF from a single preovulatory leading follicle, thereby increasing the study power and accuracy. Third, in this study, the primary endpoint is to investigate the relationship between follicular cytokine concentrations and oocyte maturity in IVF with different ovarian reserves. The result revealed that FF leptin are the best predictors of oocyte maturity among the patients with different ovarian reserves. The ART outcomes are associated with many variables including age, infertility causes, ovarian stimulation protocols, response to gonadotropins, oocyte numbers, male factor, endometrial factor, number of embryo transfer and embryo quality, except oocyte maturity. This issue is so complex over our study to answer it. Meanwhile, due to limited samples, it is difficult to run generalized linear model for the association between FF leptin concentrations and ART outcomes. Finally, it was believed that FF AMH level is positively correlated with serum AMH level. However, the approximately equal AMH levels in FF for each group were found in our results. In recent studies, serum AMH levels strongly associated with the number of oocytes, but the relationship was still ambiguous between AMH levels and oocyte quality. A main cause in most previous studies is the diverging dataset of FF investigated. For example, there was evidence that the concentrations of AMH in FF become progressively lower with increasing follicle diameters^41^. In our study, we collected and pooled the FF samples from three preovulatory follicles in each patient. Pooling the follicular fluid from multiple follicles may induce some bias and dilute the concentration of FF AMH level, which make the approximately equal AMH for each group in our study.

## Conclusion

In conclusion, the concentration of leptin in follicular fluid is closely related to ovarian reserve. The leptin level is higher in woman with normal ovarian reserve than that in woman with low or high level. Moreover, FF leptin level may serve as a biomarker to predict oocyte maturity.

## Supplementary Information

Supplementary Table 1.

## Data Availability

All data that support the findings of this study are available from the corresponding author on request.
