# Supplementary material for: The intrafollicular concentration of leptin as a potential biomarker to predict oocyte maturity in in-vitro fertilization
Source: Sci Rep. 2022 Nov 15;12:19573. doi: 10.1038/s41598-022-23737-1 (PMC9666526; doi:10.1038/s41598-022-23737-1)
Supplement: Supplementary file 1 — Supplementary Table 1. [file 41598_2022_23737_MOESM1_ESM.docx]

Supplementary table 1. LOD/LOQ and standard curve range for detected analytes.

| **Analyte** | **LoD, LoQ** |
| --- | --- |
| Serum AMH | LoD=0.010 ng/mL, LoQ=0.030 ng/mL |
| AMH in FF | LoD=0.010 ng/mL, LoQ=0.030 ng/mL |
| Basal E2 | LoD=5 pg/mL, LoQ=25 pg/mL |
| Basal LH | LoD=0.3 mIU/mL, LoQ=1 mIU/mL |
| Basal P4 | LoD=0.05 ng/mL, LoQ=0.2 ng/mL |
| Basal FSH | LoD=0.3 mlU/ml, LoQ=1 mlU/ml |
| **Analyte** | **Standard curve range** |
| PDGF-AA | 3.2–10,000 pg/mL |
| PDGF-AB/BB | 3.2–10,000 pg/mL |
| IFNr | 3.2–10,000 pg/mL |
| IL-15 | 3.2–10,000 pg/mL |
| IL-1B | 3.2–10,000 pg/mL |
| IL-6 | 3.2–10,000 pg/mL |
| IL-7 | 3.2–10,000 pg/mL |
| IL-8 | 3.2–10,000 pg/mL |
| MCP-1 | 3.2–10,000 pg/mL |
| TNFa | 3.2–10,000 pg/mL |
| EGF | 2.7 - 2,000 pg/mL |
| Angiopoitin-2 | 13.7 - 10,000 pg/mL |
| BMP-9 | 2.7 - 2,000 pg/mL |
| Endothelin-1 | 2.7 - 2,000 pg/mL |
| FGF-1 | 13.7 - 10,000 pg/mL |
| HB-EGF | 1.4 - 1,000 pg/mL |
| VEGF-C | 6.9 - 5,000 pg/mL |
| VEGF-D | 6.9 - 5,000 pg/mL |
| FGF-2 | 13.7 - 10,000 pg/mL |
| VEGF-A | 13.7 - 10,000 pg/mL |
| Leptin | 137.2 - 100,000 pg/mL |
| sFasL | 3.2–10,000 pg/mL |
| sFas | 34.3 - 25,000 pg/mL |
| Prolactin | 137.2 - 100,000 pg/mL |
| SCF | 6.9 - 5000 ng/mL |
| OPN | 548.7 - 400,000 pg/mL |
| TGFB1 | 9.8 - 2,500 pg/mL |
| TGFB2 | 9.8 - 2,500 pg/mL |
| TGFB3 | 9.8 - 2,500 pg/mL |
